# Assessment of haematological and biochemical parameters in captive green sea turtles (*Chelonia mydas*) before release

**DOI:** 10.1093/conphys/coaf069

**Published:** 2025-09-25

**Authors:** Rui Guo, Li Zhu, Xiaofei Zhai, Tongliang Wang, Jichao Wang

**Affiliations:** Ministry of Education Key Laboratory for Ecology of Tropical Islands, Key Laboratory of Tropical Animal and Plant Ecology of Hainan Province, College of Life Sciences, Hainan Normal University, No. 99, South Longkun Road, Haikou 571158, China; Ministry of Education Key Laboratory for Ecology of Tropical Islands, Key Laboratory of Tropical Animal and Plant Ecology of Hainan Province, College of Life Sciences, Hainan Normal University, No. 99, South Longkun Road, Haikou 571158, China; Ministry of Education Key Laboratory for Ecology of Tropical Islands, Key Laboratory of Tropical Animal and Plant Ecology of Hainan Province, College of Life Sciences, Hainan Normal University, No. 99, South Longkun Road, Haikou 571158, China; Ministry of Education Key Laboratory for Ecology of Tropical Islands, Key Laboratory of Tropical Animal and Plant Ecology of Hainan Province, College of Life Sciences, Hainan Normal University, No. 99, South Longkun Road, Haikou 571158, China; Ministry of Education Key Laboratory for Ecology of Tropical Islands, Key Laboratory of Tropical Animal and Plant Ecology of Hainan Province, College of Life Sciences, Hainan Normal University, No. 99, South Longkun Road, Haikou 571158, China

**Keywords:** Haematology, biochemistry, captivity, *Chelonia mydas*, conservation physiology

## Abstract

The green sea turtle (*Chelonia mydas*), a globally endangered marine reptile, faces significant population declines due to anthropogenic and environmental pressures. Captive rehabilitation programs are critical for conservation, yet captivity-induced physiological deviations may compromise post-release survival. This study establishes haematological and biochemical reference intervals for pre-release captive *C. mydas* (*n* = 40) various across juvenile, subadult, and adult life stages, and identifies key deviations from wild baselines. We found pronounced captivity-specific alterations, including elevated immature red blood cell counts and ghost cell counts in juveniles, which indicated dysregulated erythropoiesis and oxidative stress. Ontogenetic shifts revealed maladaptive macrocytic erythrocytosis in adults, likely linked to limited exercise and dietary imbalances. Biochemically, captive adults showed hyperproteinemia (total protein, 73.35 g/l) and dyslipidemia (total cholesterol, 8.98 mmol/l triglycerides, 1.53 mmol/l), indicating high-protein, high-fat diets, while hypoglucagonemia (glucose, 2.83 mmol/l) suggested compromised energy reserves. Age-dependent immune activity was observed, with juveniles exhibiting elevated leukocyte counts (19.34 × 10^9^/l), potentially due to chronic stress. These findings underscore metabolic and haematological adaptations in captivity that may hinder post-release resilience. Key biomarkers, such as immature red cell count, glucose, and lipid profiles, should guide release readiness assessments, thus ensuring rehabilitated turtles are physiologically primed for survival. This study provides a critical framework for enhancing the efficacy of sea turtle conservation translocations.

## Introduction

The green sea turtle (*Chelonia mydas*) is a globally endangered marine reptile listed on the International Union for Conservation of Nature (IUCN) Red List, with populations threatened by habitat loss, climate change, human overexploitation, and disease (Seminoff, 2023). Captive and rehabilitation programs have emerged as critical tools for *C. mydas* conservation, aiming to supplement wild populations through carefully managed releases (Kanghae *et al.,* 2021). While these initiatives provide critical care for injured or juvenile turtles, captivity inherently alters natural behaviours, diet, and activity levels, potentially inducing physiological deviations relative to wild baselines (Arena *et al.,* 2014; Pickholtz *et al.,* 2024). Such deviations may compromise post-release survival if animals are inadequately prepared for the energetic demands and environmental challenges of the wild.

Health assessment in sea turtles relies heavily on haematological and biochemical parameters, which serve as indicators of physiological status, stress, nutrition, organ function and underlying pathology (Pagano *et al.,* 2019; Jakšić *et al.,* 2022; Hayakijkosol *et al.,* 2024). Blood-based biomarkers offer a minimally invasive means to assess organ function, metabolic state, and immune competence-factors central to post-release survival. For example, plasma glucose and lipid profiles reflect nutritional adaptation (Roark *et al.,* 2009), while haematocrit and leukocyte counts indicate hydration status and inflammatory responses (Aguirre and Balazs, 2000). Serum biochemical parameters can further reveal organ dysfunction under disease stress (Esposito *et al.,* 2024). Additionally, pollutant exposure may alter immune and stress indicators, such as the neutrophil-to-lymphocyte ratio (Vaclavik *et al*., 2020). In wild *C. mydas*, baseline reference intervals for these parameters have been established in regions, such as Australia (Arthur *et al.,* 2008), Mexico (Reséndiz *et al.,* 2018) and the Bahamas (Bolten and Bjorndal, 1992). However, captive environments—characterized by controlled diets, reduced predation, and altered physical activity—can induce physiological shifts that may not align with wild baselines (Bloodgood *et al.,* 2019; Starostinetsky Malonek *et al.,* 2025).

Despite the recognized importance of physiological preparedness, a critical knowledge gap exists regarding the comprehensive evaluation of haematological and biochemical profiles in captive *C. mydas* immediately before release. For instance, captive *C. mydas* may exhibit altered lipid metabolism, with higher triglyceride and cholesterol levels attributed to diet composition (Putillo *et al.,* 2020), or reduced haematocrit due to limited exercise (Flint *et al.,* 2010). Such metabolic and haematological shifts under captivity are also diet-dependent in other aquatic species. For example, dietary phospholipid supplementation in *Pangasius nasutus* broodstock significantly modulated plasma lipid profiles and improved haematological parameters including haematocrit and haemoglobin (Torsabo *et al.,* 2024), reflecting patterns similar to those seen in captive turtles. Similarly, supplementation with *Hygrophila auriculata* enhanced growth performance and ameliorated haematological and biochemical indices in *Cirrhinus mrigala* (Kumar *et al.,* 2022), further highlighting the pivotal role of nutritional intervention in regulating metabolic and haematological health in confined aquatic organisms.

Moreover, captivity-induced stress can significantly alter both haematological and biochemical parameters, as demonstrated in various aquatic species. In *Mugil cephalus*, acclimation to captivity led to a significant reduction in red blood cells, haematocrit, haemoglobin and white blood cells, alongside increased lactate and total protein levels, indicating a stress-induced physiological response (Faggio *et al.,* 2014). Similarly, acute handling stress in *Sparus aurata* resulted in elevated glucose, lactate, and white blood cell counts, coupled with a decrease in mean corpuscular volume, underscoring the impact of husbandry practices on stress markers and haematological profiles (Fazio *et al.,* 2015). Additionally, exposure to environmental stressors, such as mercury in *Bellamya bengalensis*, caused significant reductions in haemocyte counts and protein content in the hepatopancreas and gonads, reflecting impaired immune function and metabolic disruption (Dhara *et al.,* 2022). These responses parallel the elevated markers of tissue injury or metabolic dysregulation, such as lactate dehydrogenase (LDH) and aspartate aminotransferase (AST), often observed in stressed captive turtles (Barratclough *et al.,* 2019). However, research has primarily focused on wild populations and acute health crises (e.g. cold-stunning, fibropapillomatosis). The transition from captivity to the wild imposes multiple physiological challenges, including dietary shifts, thermal fluctuations and predation stress. Evaluating whether captive-reared turtles exhibit biochemical profiles consistent with wild counterparts is essential for predicting post-release fitness. Without standardized pre-release assessments, however, it remains unclear which metrics best predict successful adaptation.

This study aims to establish haematological and biochemical reference intervals for captive *C. mydas* during the pre-release phase. By analysing data from rehabilitated turtles across multiple captive facilities, this study aims to characterize physiological variations associated with captivity and identify biomarkers of nutritional adequacy and stress. The results will enhance the scientific rigour of conservation translocations, ensuring that captive-bred turtles are physiologically primed for wild survival.

## Materials and methods

### Animal ethics

All experiments were carried out according to ‘Guidance of the Care and Use of Laboratory Animals’ approved by Animal Research Ethics Committee of Hainan Provincial Education Center for Ecology and Environment, Hainan Normal University, China (202420).

### Animals

Blood samples were collected from a total of 40 *C. mydas* before their release. The group included 9 juveniles, 29 subadults and 2 adults sourced from the Turtle Rescue Station in Qionghai, China, and the Guangdong Huihai Hawksbill Sea Turtle National Nature Reserve. For each individual, physiological measurements were recorded, including curved carapace length (CCL) and width (CCW). Turtles were categorized into three life-stage groups (juveniles, subadults, adults) using established midline CCL criteria, juveniles were defined as CCL ≤ 30 cm, subadults as CCL > 30 cm and ≤ 75 cm, and adults as CCL > 75 cm (Broderick *et al.,* 2003; Cardona *et al.,* 2010).

### Blood collection and analysis

Following cutaneous disinfection of the jugular venipuncture site with iodophor, 0.5 ml of blood was aseptically collected from the external jugular vein using a 5-ml syringe. 200 μl of the whole blood sample was aliquoted into an EDTA K₂ tube for complete blood count analysis. 300 μl of the whole blood sample was aliquoted into a serum separator tube/clot activator tube (e.g. Seamaty biochemistry tube) for biochemical analysis. Complete blood count parameters were measured within 2 hours of collection using an automated haematology analyser. Analysed parameters included: 1) leukocyte profile: white blood cell count (WBC), absolute count and percentage of heterophils and eosinophils, lymphocytes, monocytes and basophils. 2) Erythrocyte indices: red blood cell count (RBC), haemoglobin concentration (HGB), haematocrit (HCT), mean corpuscular volume (MCV), mean corpuscular haemoglobin (MCH), mean corpuscular haemoglobin concentration (MCHC), red cell distribution width (RDW-SD, RDW-CV), absolute immature red cell count (IRBC), percentage of immature red cells, abnormally shaped red blood cell count, also known as ghost cell count. 3) Thrombocyte parameters: thrombocyte count, absolute clumped thrombocyte count and clumped thrombocyte percentage. Serum biochemical analyses were performed using an automated chemistry analyser. The assayed analytes included: (i) Proteins: albumin, total protein, globulin, albumin/globulin ratio. (ii) Liver and enzymes: total bilirubin, gamma-glutamyl transferase (GGT), aspartate aminotransferase (AST), alanine aminotransferase (ALT), alkaline phosphatase (ALP), total bile acids (TBA), amylase, lipase, lactate dehydrogenase (LDH). (iii) Muscle enzymes and metabolites: creatine kinase, creatinine, uric acid, urea. (iv) Metabolism: glucose, total cholesterol, triglycerides. (v) Electrolytes and minerals: total carbon dioxide (TCO₂), calcium (Ca), inorganic phosphorus (PHOS). (vi) Calculated ratios: aspartate aminotransferase to alanine aminotransferase ratio (AST/ALT), calcium-phosphorus product (Ca^*^PHOS).

### Statistical analysis

All data were analysed using SPSS 19.0 (IBM Corp., Armonk, NY, USA). Continuous variables are presented as mean ± standard deviation (mean ± SD), while categorical variables are expressed as frequencies and percentages. One-way analysis of variance (ANOVA) was used to compare continuous variables across developmental stages (juveniles, subadults and adults). Homogeneity of variance was assessed using Levene's test. When variance was homogeneous (*P* > 0.05), multiple comparisons were performed using the least significant difference (LSD) post-hoc test. If the assumption of homogeneity was violated (*P* < 0.05), Dunnett’s T3 test was applied instead. All statistical tests were two-tailed, with a significance level set at 0.05.

## Results

This study categorized *C. mydas* into three age groups (juveniles, subadults and adults) based on their CCL. The results showed that juveniles exhibited the smallest CCL and CCW (CCL, 13.03 ± 1.86 cm; CCW, 11.79 ± 1.16 cm). Subadults showed intermediate values (CCL, 50.10 ± 9.59 cm; CCW, 44.69 ± 8.00 cm). Adults displayed the largest carapace dimensions (CCL, 79.00 ± 7.07 cm; CCW, 68.95 ± 14.21 cm).

The haematological analysis of captive *C. mydas* revealed significant variations in leukocyte and erythrocyte parameters across different age groups (juveniles, subadults and adults). For leukocytes, juvenile *C. mydas* exhibited significantly higher WBC counts (19.34 ± 1.66) × 10^9^/l compared to subadults (12.69 ± 4.72) × 10^9^/l and adults (7.45 ± 0.67) × 10^9^/l (*P* < 0.01), with all groups falling within the reference range (3.64–28.42) × 10^9^/l (Table 1). Lymphocytes were markedly elevated in juveniles (10.34 ± 2.74) × 10^9^/l but decreased progressively in subadults (4.98 ± 2.42) × 10^9^/l and adults (2.22 ± 0.50) × 10^9^/l (*P* < 0.01), remaining within the reference range (0.08–9.67) × 10^9^/l (Table 1). Heterophils and eosinophils were highest in juveniles (7.50 ± 4.44) × 10^9^/l and declined with age (*P* < 0.05), though all stages were within the reference limits (0.00–14.83) × 10^9^/l (Table 1).

**Table 1:** Haematological values of captive *C. mydas*

	**Juveniles**		**Subadults**		**Adults**		**Reference range**
	**Mean ± SD**	**Range**	**Mean ± SD**	**Range**	**Mean ± SD**	**Range**	
**WBC (10** ^**9**^**/l)**	19.34 ± 1.66a	18.2–21.77	12.69 ± 4.72b	1.89–22.10	7.45 ± 0.67b	6.97–7.92	3.64–28.42
**Heterophils and eosinophils (10** ^**9**^**/l)**	7.50 ± 4.44	4.35–13.94	6.73 ± 2.88	1.00–11.98	4.91 ± 0.04	4.88–4.93	0.00–14.83
**Lymphocytes (10** ^**9**^**/l)**	10.34 ± 2.74a	6.47–12.78	4.98 ± 2.42b	0.44–9.86	2.22 ± 0.50b	1.86–2.57	0.08–9.67
**Monocytes (10** ^**9**^**/l)**	0.98 ± 0.11	0.81–1.05	0.63 ± 0.54	0.02–2.26	0.21 ± 0.21	0.06–0.35	0.00–2.29
**Basophils (10** ^**9**^**/l)**	0.52 ± 0.25	0.18–0.79	0.35 ± 0.27	0.03–1.00	0.13 ± 0.01	0.12–0.13	0.00–7.80
**Percentage of heterophils and eosinophils (%)**	37.62 ± 18.48a	23.69–64.04	53.73 ± 14.37b	13.36–77.53	66.17 ± 6.46b	61.60–70.74	10.17–74.15
**Percentage of lymphocytes (%)**	54.55 ± 17.61a	29.72–69.64	38.50 ± 11.73b	14.36–63.64	29.56 ± 4.12b	26.65–32.47	6.25–56.82
**Percentage of monocytes (%)**	5.12 ± 0.93	3.74–5.70	5.05 ± 5.33	0.89–29.72	2.63 ± 2.58	0.80–4.45	0.00–13.40
**Percentage of basophils (%)**	2.71 ± 1.40	0.96–4.35	2.73 ± 1.90	0.59–7.97	1.64 ± 0.23	1.48–1.80	5.69–43.17
**RBC (10** ^**12**^**/l)**	0.73 ± 0.11	0.57–0.80	0.61 ± 0.18	0.29–0.92	0.61 ± 0.18	0.48–0.73	0.21–0.71
**HGB (g/l)**	100.37 ± 16.85	78.09–117.30	109.14 ± 35.78	50.37–168.77	109.97 ± 32.74	86.82–133.12	27.17–99.53
**HCT (%)**	31.07 ± 5.26	26.07–36.44	38.85 ± 13.08	16.98–60.61	40.69 ± 9.18	34.20–47.18	11.20–37.20
**MCV (fL)**	427.62 ± 64.83a	332.69–478.12	632.99 ± 59.55b	509.29–764.00	679.97 ± 52.48b	642.86–717.08	458.91–589.55
**MCH (pg)**	136.97 ± 8.97a	124.86–146.55	178.07 ± 12.79b	147.38–203.11	181.70 ± 0.47b	181.37–182.03	102.86–172.93
**MCHC (g/l)**	324.38 ± 36.74a	288.45–375.32	281.32 ± 17.72b	247.75–311.32	267.99 ± 19.20b	253.85–282.13	281.75–455.61
**RDW-SD (fL)**	241.25 ± 29.48	215.00–278.00	219.61 ± 30.03	173.00–319.00	192.00 ± 59.40	150.00–234.00	82.71–130.27
**RDW-CV (%)**	20.78 ± 0.50	20.22–21.37	19.11 ± 2.19	14.98–23.03	16.28 ± 3.36	13.90–18.65	7.48–13.53
**IRBC (10** ^**9**^**/l)**	2.83 ± 2.07a	1.55–5.91	1.08 ± 1.20b	0.06–4.23	0.21 ± 0.25b	0.03–0.38	0.00–0.51
**Percentage of IRBC (%)**	0.15 ± 0.10	0.08–0.30	0.08 ± 0.09	0.00–0.36	0.02 ± 0.02	0.00–0.03	0.00–0.11
**Ghost cell count (10** ^**9**^**/l)**	43.75 ± 42.28a	4.90–102.50	14.75 ± 12.25b	0.30–40.10	7.83 ± 9.74b	0.94–14.72	0.00–0.32
**Ghost cell percentage (%)**	6.84 ± 7.78	0.61–18.10	3.19 ± 3.27	0.03–11.54	1.61 ± 2.09	0.13–3.09	0.00–0.07
**Thrombocytes (10** ^**9**^**/l)**	4.66 ± 1.92	2.23–6.46	3.75 ± 2.61	0.44–9.90	1.00 ± 0.69	0.51–1.49	0.00–12.40
**Clumped thrombocytes (10** ^**9**^**/l)**	0.05 ± 0.09	0.00–0.18	0.02 ± 0.05	0.00–0.18	0.03 ± 0.04	0.00–0.06	0.00–0.29
**Percentage of clumped thrombocyte (%)**	0.81 ± 1.28	0.00–2.69	0.49 ± 1.35	0.00–6.43	1.91 ± 2.70	0.00–3.82	0.00–13.35

The letters a, b and c indicate significant differences at the *P* < 0.05 level. WBC: white blood cell count; RBC: red blood cell count; HGB: haemoglobin concentration; HCT: haematocrit; MCV: mean corpuscular volume; MCH: mean corpuscular haemoglobin; MCHC: mean corpuscular haemoglobin concentration; RDW-SD: red cell distribution width-standard deviation; RDW-CV: red blood cell distribution width-coefficient of variation; IRBC: immature red cell count.

In erythrocyte parameters, RBC were consistent across subadults and adults but differed from juveniles (juveniles, (0.73 ± 0.11) × 10^12^/l; subadults, (0.61 ± 0.18) × 10^12^/l; adults, (0.61 ± 0.18) × 10^12^/l) (Table 1). While HGB and HCT values increased with maturity (e.g. HGB: juveniles, 100.37 ± 16.85 g/l; subadults, 109.14 ± 35.78 g/l; adults, 109.97 ± 32.74 g/l. HCT: juveniles, 31.07 ± 5.26%; subadults, 38.85 ± 13.08%; adults, 40.69 ± 9.18%) (Table 1). Notably, MCV and MCH rose significantly from juveniles to adults (MCV, 427.62 ± 64.83 fL vs. 679.97 ± 52.48 fL, *P* < 0.01; MCH, 136.97 ± 8.97 pg vs. 181.70 ± 0.47 pg, *P* < 0.01), reflecting larger and more haemoglobin-rich erythrocytes in mature individuals. IRBC were elevated in juveniles (2.83 ± 2.07) × 10^9^/l but declined sharply in adults (0.21 ± 0.25) × 10^9^/l (*P* < 0.05), with juvenile values surpassing the reference range (0.00–0.51) × 10^9^/l (Table 1), indicating reduced erythropoietic activity in adulthood. Ghost cell count was highest in juveniles (43.75 ± 42.28) × 10^9^/l and decreased with age (*P* < 0.05), far exceeding the reference range (0.00–0.32) × 10^9^/l in all stages (Table 1). These findings highlight age-dependent haematological adaptations, likely linked to physiological maturation and metabolic demands in *C. mydas*.

The biochemical analysis of captive *C. mydas* revealed significant age-related variations in liver, renal, cardiac, and pancreatic homeostasis markers. Liver function parameters, including albumin and total protein, exhibited progressive increases with age (juveniles: albumin, 12.93 ± 1.49 g/l; total protein, 32.42 ± 12.62 g/l; subadults: albumin, 21.06 ± 2.47 g/l; total protein, 57.05 ± 5.60 g/l; adults: albumin, 26.95 ± 4.60 g/l; total protein, 73.35 ± 15.49 g/l) (*P* < 0.01), with adult total protein exceeding the reference range (26–68 g/l) (Table 2). AST remained stable across age groups (222.40–258 U/l), within the reference range (66–315 U/l), while ALT spiked in adults (102.50 ± 136.47 U/l), suggesting potential age-dependent hepatic stress (Table 2). Globulin levels were lower in juveniles (24.88 ± 3.23 g/l) compared to subadults (36.00 ± 5.07 g/l) and adults (46.35 ± 10.96 g/l) (*P* < 0.01), with adults falling within the reference range (26–45 g/l) (Table 2). Total bilirubin and GGT were consistently low across all groups, with GGT levels below 2 U/l in all age categories (Table 2).

**Table 2:** Biochemical values of captive *C. mydas*

	**Juveniles**		**Subadults**		**Adults**		**Reference range**
	**Mean ± SD**	**Range**	**Mean ± SD**	**Range**	**Mean ± SD**	**Range**	
**Albumin (g/l)**	12.93 ± 1.49a	10.70–13.80	21.06 ± 2.47b	14.00–25.10	26.95 ± 4.60c	23.70–30.20	10–25
**Total protein (g/l)**	32.42 ± 12.62a	11.00–42.20	57.05 ± 5.60b	45.30–70.50	73.35 ± 15.49c	62.40–84.30	26–68
**Globulin (g/l)**	24.88 ± 3.23a	20.80–28.40	36.00 ± 5.07b	28.20–52.30	46.35 ± 10.96c	38.60–54.10	26–45
**Albumin/Globulin**	0.52 ± 0.03	0.49–0.56	0.60 ± 0.09	0.27–0.73	0.59 ± 0.04	0.56–0.61	
**Total bilirubin (μmol/l)**	4.83 ± 2.72	2.30–7.70	8.28 ± 6.31	0.30–23.80	2.00	2.00	
**GGT (U/l)**	<2		<2		<2		
**AST (U/l)**	222.40 ± 78.61	92.00–279.00	229.25 ± 85.57	113.00–538.00	258	258	66–315
**ALT (U/l)**	40.20 ± 22.37a	18.00–76.00	12.49 ± 4.76a	7.00–27.00	102.50 ± 136.47b	6.00–199.00	
**ALP (U/l)**	174.80 ± 101.78a	25.00–293.00	39.37 ± 12.25b	17.00–75.00	36.50 ± 12.02b	28.00–45.00	24–48
**TBA (μmol/l)**	<1		<1		<1		
**Amylase (U/l)**	733.00 ± 523.09a	428.00–1337.00	1184.96 ± 348.15b	743.00–2242.00	1139.00 ± 651.95b	678.00–1600.00	
**Lipase (U/l)**	17.80 ± 7.39	8.00–27.00	17.75 ± 3.34	11.00–24.00	17.00 ± 4.24	14.00–20.00	
**LDH (U/l)**	577.80 ± 368.35	112.00–1057.00	388.43 ± 180.02	212.00–1158.00	501.50 ± 23.33	485.00–518.00	100–800
**Creatine kinase (U/l)**	1272.40 ± 1305.04	162.00–2907.00	2352.00 ± 1197.42	475.00–3932.00			
**Creatinine (μmol/l)**	25.24 ± 8.16	14.10–35.90	22.88 ± 7.91	12.30–46.70	13.45 ± 3.46	11.00–15.90	10–25
**Uric acid (μmol/l)**	41.18 ± 30.46	13.33–84.94	52.86 ± 28.20	21.87–112.37	45.92		15–107
**Urea (mmol/l)**	14.88 ± 4.12	9.93–20.38	16.90 ± 6.60	6.41–29.87	7.98 ± 7.12	2.94–13.01	12.5–39.3
**Urea/Creatinine**	700.03 ± 362.64	276.75–1249.56	777.28 ± 397.10	273.00–1786.00	685.90 ± 708.19	185.13–1186.66	
**Glucose (mmol/l)**	6.97 ± 1.03a	5.34–8.10	5.15 ± 1.00b	3.79–7.20	2.83 ± 0.50c	2.47–3.18	4–6.95
**Total cholesterol (mmol/l)**	5.32 ± 2.72	0.98–7.82	5.82 ± 1.59	1.90–8.62	8.98 ± 3.70	6.36–11.59	3.88–11.5
**Triglycerides (mmol/l)**	0.42 ± 0.16	0.31–0.53	1.31 ± 0.77	0.44–3.19	1.53 ± 0.74	1.00–2.05	0.3–2.94
**TCO**_**2**_ **(mmol/l)**	18.78 ± 3.04	16.10–23.50	19.47 ± 2.14	16.50–24.80	19.45 ± 0.35	19.20–19.70	
**Ca (mmol/l)**	1.62 ± 0.29	1.27–2.03	1.74 ± 0.14	1.33–1.96	1.89 ± 0.14	1.79–1.99	1.25–3.5
**PHOS (mmol/l)**	2.43 ± 1.50	0.54–4.19	2.33 ± 0.44	1.69–3.40	2.50 ± 1.23	1.63–3.37	1.77–3.06
**AST/ALT**	5.98 ± 1.74	3.67–8.11	20.52 ± 8.98	9.39–42.71	46.86		
Ca^*^PHOS	3.68 ± 1.98	0.95–6.20	4.07 ± 0.89	2.58–6.11	4.64 ± 1.98	3.24–6.04	

The letters a, b and c indicate significant differences at the *P* < 0.05 level. GGT: gamma-glutamyl transferase; AST: aspartate aminotransferase; ALT: alanine aminotransferase; ALP: alkaline phosphatase; TBA: total bile acids; LDH: lactate dehydrogenase; TCO₂: total carbon dioxide; Ca: calcium; PHOS: inorganic phosphorus.

Renal function markers like creatinine declined with maturity (juveniles, 25.24 ± 8.16 μmol/l; subadults, 22.88± 7.91 μmol/l; adults, 13.45 ± 3.46 μmol/l), aligning with the reference range (10–25 μmol/l), whereas urea dropped below the reference (12.5–39.3 mmol/l) in adults (7.98 ± 7.12 mmol/l) (Table 2). Cardiac enzymes such as creatine kinase were elevated in subadults (2352.00 ± 1197.42 U/l) but undetectable in adults, while LDH showed no significant age-related trends (Table 2). Glucose metabolism demonstrated a marked decline with age (juveniles, 6.97 ± 1.03 mmol/l; subadults, 5.15 ± 1.00 mmol/l; adults, 2.83 ± 0.50 mmol/l), falling below the reference range (4–6.95 mmol/l) in adults (Table 2). Lipid profiles revealed higher total cholesterol and triglycerides in adults (total cholesterol, 8.98 ± 3.70 mmol/l; triglycerides, 1.53 ± 0.74 mmol/l), within reference limits (total cholesterol, 3.88–11.5 mmol/l; triglycerides, 0.3–2.94 mmol/l) (Table 2). Pancreatic enzymes like amylase peaked in subadults (1184.96 ± 348.15 U/l), whereas lipase remained stable across groups (Table 2). Ca levels also rose with age, from 1.62 ± 0.29 mmol/l in juveniles to 1.89 ± 0.14 mmol/l in adults, within the reference range (1.25–3.5 mmol/l) (Table 2). Notably, GGT and TBA were undetectable (<2 U/l and < 1 μmol/l, respectively) in all groups.

These findings underscore ontogenetic shifts in biochemical homeostasis, reflecting adaptive metabolic and physiological changes during maturation in captive *C. mydas*.

## Discussion

This study provides a comprehensive assessment of haematological and biochemical parameters in captive *C. mydas* across life stages before release, revealing significant ontogenetic (age-dependent) shifts across multiple physiological parameters, highlighting the dynamic nature of haematopoiesis and metabolic homeostasis throughout development in captivity. These findings establish critical pre-release baselines and further identify deviations from wild physiological norms, underscoring adaptations to the captive environment. Collectively, the results offer vital insights for ensuring release readiness and inform key considerations for the success of conservation translocation efforts.

### Haematological adaptations and deviations in captive *C. mydas*

Our findings reveal pronounced captivity-specific alterations in the erythropoietic system and erythrocyte integrity across the ontogeny of *C. mydas*. Juvenile captives exhibited significantly elevated IRBC compared to established wild reference values, surpassing values reported in previous captive/wild comparisons (Flint *et al.,* 2010; Reséndiz *et al.,* 2018; Bloodgood *et al.,* 2019). This pronounced elevation in IRBC is indicative of accelerated yet potentially dysregulated erythropoietic activity in juveniles, likely induced by captivity-associated physiological stressors such as dietary imbalances, constrained space, or suboptimal environmental parameters (Agustina *et al.,* 2020). These factors contrast sharply with the stable haematopoiesis inferred for wild juveniles utilizing natural foraging habitats (Reséndiz *et al.,* 2018). A particularly striking finding across all captive age groups was the extreme elevation of ghost cell count, reaching up to 43.75 × 10^9^/l, vastly exceeding the established wild baseline range (0–0.32 × 10^9^/l) (Flint *et al.,* 2010). This pervasive elevation strongly suggests sustained oxidative stress and/or recurrent subclinical haemolysis resulting from continuous exposure to the captive milieu, a hypothesis corroborated by evidence of lipid peroxidation in captive cheloniids (Barratclough *et al.,* 2019).

The environment in captivity significantly affected erythrocyte maturation. While MCV and MCH increased with age in captives, mirroring an essential ontogenetic pattern observed in wild populations (Bloodgood *et al.,* 2019), this shift was accelerated and exaggerated. Crucially, captive individuals developed a prominent macrocytic erythrocytosis characterized by a pronounced steep rise in MCV from juveniles (428 fL), consistently exceeding the range reported for large wild juveniles to adults (53–70 fL; Reséndiz *et al.,* 2018). This maladaptive deviation, absent in free-ranging conspecifics, likely stems from two key captivity-related factors: 1) limited swimming space suppressing exercise-induced erythropoietin signalling essential for erythrocyte regulation (Weng *et al.,* 2021), and 2) nutritional deficiencies inherent in formulated diets, particularly concerning micronutrients critical for balanced erythropoiesis and haemoglobin synthesis. This nutritional component is further suggested by the observed higher MCHC in captive adults compared to their wild counterparts, potentially reflecting altered haemoglobin packaging linked to dietary iron bioavailability (Reséndiz *et al.,* 2018; Agustina *et al.,* 2020). Simultaneously, functional compromises were observed. Juvenile captives displayed a trend towards sub-optimal HCT compared to representative wild values (Fernández-Sanz *et al*., 2025).

Collectively, these hematologic disruptions—accelerated but dysregulated juvenile erythropoiesis, profound evidence of oxidative stress/haemolysis, maladaptive macrocytic erythrocytosis with age, suboptimal HCT in juveniles, and thrombocyte deficiency in adults—point towards a compromised oxygen transport capacity and reduced wound-healing potential in captive *C. mydas*. This multi-factorial impact underlines the physiological challenges associated with recreating optimal conditions for marine turtles in long-term captivity.

Furthermore, the age-related progressive decline in WBC observed in sea turtles (juveniles > subadults > adults) aligns with patterns in wild populations, reflecting reduced immune activity as individuals mature (Work and Balazs, 1999). However, captive juveniles exhibit significantly elevated WBC levels compared to wild conspecifics (Zwarg *et al.,* 2014), likely attributable to chronic stress or subclinical infections in controlled environments (Starostinetsky Malonek *et al.,* 2025). While increased lymphocyte activity in juveniles corresponds to age-specific immune maturation in wild settings, the persistently high WBC in captivity suggests chronic low-grade inflammation. This may stem from captive conditions, heightened stress responses, and diminished microbial exposure (Aguirre and Balazs, 2000).

### Biochemical signatures of captive nutrition and metabolic adaptation

The biochemical profiles of captive *C. mydas* reveal significant metabolic adaptations induced by controlled diets and reduced activity levels. Notably, captive adults exhibit hyperproteinemia, with total protein and albumin concentrations exceeding wild baselines recorded in Mexican populations (Labrada-Martagón *et al.,* 2010; Reséndiz *et al.,* 2018). This elevation is strongly associated with the high-protein composition of captive diets, which contrasts with the mixed seagrass-algae diet of wild conspecifics and may induce hepatic overactivity (McMaster *et al.,* 2006; Reséndiz *et al.,* 2018). While elevated globulin could reflect immune stimulation due to exposure to captive pathogens, the albumin/globulin ratio remains within wild reference ranges, suggesting preserved immune homeostasis (Aguirre and Balazs, 2000). These findings align with observations in other captive *C. mydas* populations, where protein-rich diets similarly elevate total protein, underscoring the need for dietary adjustments to better approximate wild nutritional profiles (Bloodgood *et al.,* 2019; Sinaei *et al.,* 2019).

Captive conditions also markedly alter lipid metabolism, with adults exhibiting elevated triglycerides and total cholesterol compared to wild foragers (Bolten and Bjorndal, 1992). Similar lipid dysregulation has been documented in captive *Lepidochelys kempii*, where lipid-rich feeds disrupt homeostasis and increase risks of hepatic lipidosis and impaired vascular function (McMaster *et al.,* 2006; Anderson *et al.,* 2011). Wild *C. mydas*, which primarily consume low-fat seagrasses, maintain significantly lower lipid levels, reinforcing the necessity of reducing dietary fat in pre-release conditioning protocols to mitigate metabolic imbalances that could compromise post-release foraging efficiency (Sinaei *et al.,* 2019). Renal parameters exhibit an age-dependent pattern, with urea levels significantly higher in juveniles and subadults than in captive adults. These juvenile/subadult values exceed the lower thresholds of wild baselines, potentially indicating subclinical dehydration or renal stress due to osmotic imbalances in captivity (Anderson *et al.,* 2011; Sinaei *et al.,* 2019).

A particularly concerning finding is the hypoglucagonemia observed in captive adults, which falls below wild reference intervals (Roark *et al.,* 2009; Sinaei *et al.,* 2019; Li and Chang, 2020). This suppressed glucose state may reflect reduced glycogen reserves or prolonged fasting, raising concerns about energy availability for post-release migrations. Wild turtles maintain higher glucose levels to support active foraging and long-distance movements (March *et al.,* 2019), suggesting that captive individuals may be metabolically ill-prepared for the energetic demands of reintroduction. Similarly, liver and pancreatic enzymes exhibit age-specific variations, with ALP decreasing with maturity, consistent with wild ontogenetic trends (Reséndiz *et al.,* 2018). Conversely, AMY in subadults, exceeding wild levels, likely due to carbohydrate-rich captive diets (Bloodgood *et al.,* 2019; Sinaei *et al.,* 2019).

Collectively, these findings highlight the profound metabolic consequences of captivity in *C. mydas*, driven by dietary mismatches and reduced activity. To enhance the success of reintroduction programs, captive diets should be optimized to better replicate wild nutritional profiles, with particular attention to protein and lipid content. Additionally, pre-release physiological assessments should evaluate glucose metabolism and renal function to ensure that individuals are metabolically prepared for the challenges of wild foraging and migration.

### Limitations and conservation recommendations

Despite a limited adult sample size (*n* = 2) constraining broad generalizations, the observed trends correspond with wider evidence of captivity-induced physiological shifts in reptiles (Flint *et al.,* 2010). Future research must prioritize longitudinal monitoring of released cohorts to establish definitive links between pre-release biochemical markers and post-release survival outcomes. Critically, we propose that targeted enrichment strategies (e.g. large swim tanks, foraging puzzles) designed to simulate natural activity levels could mitigate haematological and metabolic derangements, an approach validated in sea turtle rehabilitation settings (Pickholtz *et al.,* 2024). Concurrent nutritional optimization—specifically, reducing dietary protein content while increasing indigestible fibre to emulate natural seagrass consumption (Putillo *et al.,* 2020)—promises to normalize serum lipid profiles and support renal function. The urgent implementation of such evidence-based pre-release conditioning protocols is paramount.

## Conclusion

Captive *C. mydas* show haematological and biochemical changes that may reduce post-release survival, including erythrocytic macrocytosis, elevated plasma lipids, and juvenile lymphocytosis. Metabolic adaptations like hyperproteinemia and dyslipidemia likely disrupt energy allocation for reproduction and foraging, while diet-induced shifts may hinder readaptation to wild herbivory. Critical release biomarkers include IRBC and glucose, with diet-sensitive parameters (total protein and triglycerides) requiring pre-release correction. Proactive dietary management and physiological monitoring are vital to enhance reintroduction success.

## Supplementary Material

Web_Material_coaf069

## Data Availability

The data underlying this article are available in the article and in its online supplementary material.
